# Antiretroviral Treatment Program Retention among HIV-Infected Children in the Democratic Republic of Congo

**DOI:** 10.1371/journal.pone.0113877

**Published:** 2014-12-26

**Authors:** John Ditekemena, Christophe Luhata, William Bonane, Modeste Kiumbu, Antoinette Tshefu, Robert Colebunders, Olivier Koole

**Affiliations:** 1 Elizabeth Glaser Paediatric AIDS Foundation, Kinshasa, Democratic Republic of Congo; 2 Kinshasa School of Public Health, Kinshasa, Democratic Republic of Congo; 3 Heal Africa, Goma, Democratic Republic of Congo; 4 University of Antwerp, Antwerp, Belgium; 5 London School of Hygiene and Tropical Medicine, London, United Kingdom; Faculty of Medicine, Australia

## Abstract

**Background:**

Retaining patients with HIV infection in care is still a major challenge in sub- Saharan Africa, particularly in the Democratic Republic of Congo (DRC) where the antiretroviral treatment (ART) coverage is low. Monitoring retention is an important tool for evaluating the quality of care.

**Methods and Findings:**

A review of medical records of HIV -infected children was performed in three health facilities in the DRC: the Amo-Congo Health center, the Monkole Clinic in Kinshasa, and the HEAL Africa Clinic in Goma. Medical records of 720 children were included. Kaplan Meier curves were constructed with the probability of retention at 6 months, 1 year, 2 years and 3 years. Retention rates were: 88.2% (95% CI: 85.1%–90.8%) at 6 months; 85% (95% CI: 81.5%–87.6%) at one year; 79.4% (95%CI: 75.5%–82.8%) at two years and 74.7% (95% CI: 70.5%–78.5%) at 3 years. The retention varied across study sites: 88.2%, 66.6% and 92.5% at 6 months; 84%, 59% and 90% at 12 months and 75.7%, 56.3% and 85.8% at 24 months respectively for Amo-Congo/Kasavubu, Monkole facility and HEAL Africa. After multivariable Cox regression four variables remained independently associated with attrition: study site, CD4 cell count <350 cells/µL, children younger than 2 years and children whose caregivers were member of an independent church.

**Conclusions:**

Attrition remains a challenge for pediatric HIV positive patients in ART programs in DRC. In addition, the low coverage of pediatric treatment exacerbates the situation of pediatric HIV/AIDS.

## Introduction

According to the UNAIDS 2013 report, worldwide 3.3 million children under 15 years were living with HIV in 2012. Over 90% of these infected children live in sub-Saharan Africa [1]. It was estimated that during the same year 260,000 new HIV infections occurred among children, most of them as a result of mother-to-child transmission of HIV (MTCT) [1]. Mother-to-child transmission rates declined overall from an estimated 26% in 2009 to 17% in 2012, and coverage of prevention of mother -to-child transmission (PMTCT) services increased to 65%. The number of children younger than 15 years receiving ART increased to 34% [1]. However, these summary estimates comprise enormous disparities between the different countries [1]. In 2012, the coverage of PMTCT services in the Democratic Republic of Congo (DRC) was estimated to be only 13%, and only 9% of children younger than 15 years in need of ART (according to the WHO 2010 guidelines) were receiving ART [2], [3].

Evidence shows that early administration of ART in HIV-infected infants reduces mortality and HIV progression by 75% [4]. However, achieving this depends on several factors, including the early diagnosis of HIV infection, adequate quality of antiretroviral (ARV) drug prescription, good support for children on ART and maintaining/retaining these HIV-infected children in care and ART programs [4], [5].

Patients' retention in care programs remains a major challenge in sub- Saharan Africa [6]–[10]. It is also one of the key indicators to assess the success of ART programs [6].

Retention rates among children vary from 71 to 95% at one year, and between 62 and 93% at 2 years in sub-Saharan Africa [5].

To maximize retention in ART programs, a number of factors need to be considered: structural factors such as transportation, availability and accessibility of services, service providers' attitude, caregiver's HIV status and religious and other beliefs (traditional healers), and patient-related factors [11], [12].

The objective of this study was to determine retention rates of HIV-infected children on ART in three different programs and sites in the DRC, and to assess risk factors for attrition. Based on the study findings, we propose recommendations to improve retention in pediatric HIV care and treatment programs.

## Methods

### Study Sites

**Figure 1:**
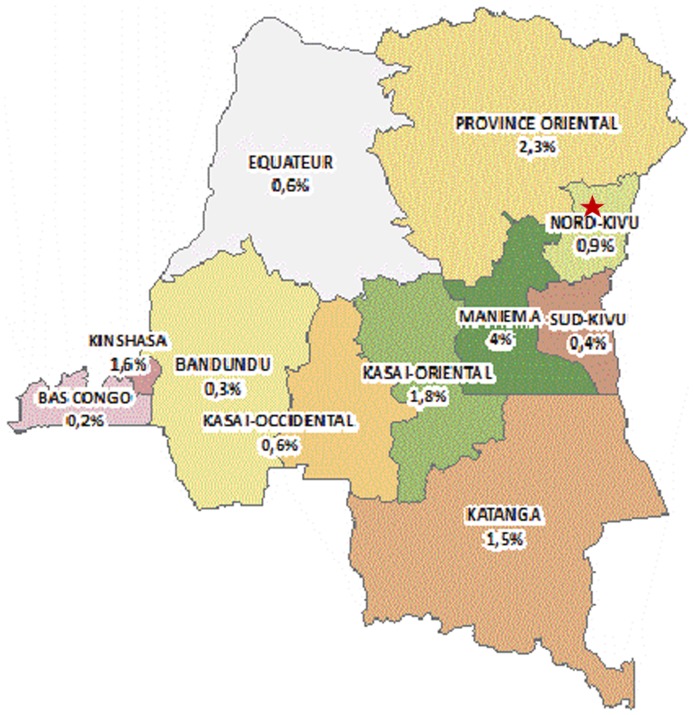
Map of the Democratic Republic of Congo with provincial HIV prevalence.

Three facilities, known to offer care for HIV infected children and with a sufficient number of patients were asked and agreed to participate in the study. Two of these facilities are located in Kinshasa, the capital of the DRC (an Amo-Congo health facility and the Centre Hospitalier Monkole). The third facility (HEAL Africa) is located in Goma, the capital city in the North Kivu province (**Fig. 1**). Amo-Congo is a Congolese non-governmental organization (NGO) that began in 1993 with providing support for orphans and vulnerable children in Kinshasa. Over the years this NGO expanded its activities to several provinces and included voluntary counseling and testing, and provision of HIV care to adults and children. In 2005 Amo-Congo started, with financial support from Global Fund for AIDS Tuberculosis and Malaria (GFATM) with the provision of ART in their Ambulatory Treatment Centers (ATCs), which are standalone clinics that offer counseling and testing, and HIV care. This study was conducted at the ATC in Kasa-vubu in Kinshasa.

The “Centre Hospitalier Monkole”, the Monkole Hospital, is a private health hospital (55 beds), in the Mount Ngafula I health zone in Kinshasa which started its activities in 1991. Their HIV unit started activities in 2005 with financial support from GFATM through the United Nations Development Programme (UNDP), and organizes HIV activities such as HIV prevention activities, ART and management of opportunistic infections. Both the Amo-Congo and Monkole health facility serve an urban population.

HEAL Africa Hospital was started in 1998 and is located in Goma/North- Kivu, and serves as a center of excellence for the province. It has 155 beds and works with 4 services/departments including surgery, internal medicine, pediatrics and obstetrics and gynecology. The HIV/AIDS pediatric care service of this facility is called AIDS Children Program (CAP), and was integrated into HEAL Africa hospital in 2005 (with financial support from UNICEF, Global Strategies and the Clinton Foundation). This facility serves populations from peri-rural and rural areas, and supports community organization in the rural area.

### Study type and inclusion criteria

From October 2012 to August 2013, we conducted a retrospective cohort study of HIV-infected children on ART at these three health facilities in the DRC.

Children older than 6 months and younger than 15 years at the moment of data collection who had initiated ART at least three months prior to data collection were included in the study.

### Data collection

We conducted a retrospective chart review of 720 medical charts. In each of the three research sites a random sample of medical charts was selected from a sampling frame that consisted of all children ever started on ART at that site between September 2007 and May 2012. The sampling frame was prepared by the site supervisor; the list of the medical files to be selected was generated by the data analyst using a computerized random number generator (‘sample’ function in Stata). Randomly selected children that were not eligible for the study were replaced by the next child in the sampling frame. Only one child per family was included in the study (the first child from the family on the list).

A specifically designed study form was used for data abstraction, which was carried out by a team of two nurses. All the members of the study team were trained in ethics (for confidentiality issues) and data abstraction.

### Data analysis and sample size

A random sample of 250 ART-treated children in each site was needed to allow the estimation of the retention rate to be measured with a precision of 5%, assuming the retention rate was at least 80%. In all three study sites, patients received ARV medication according to the WHO 2010 guidelines [3]. Once on ART, HIV-positive children were required to come to the clinic every three months. The child or the caregiver/guardian had to go to the pharmacy every month to collect the ARV drugs. A child or caregiver visiting the clinic within the three months preceding the data collection was considered to be retained in the program.

Attrition was defined as death or loss to follow-up. Lost to follow up stands for a person under treatment and missing the scheduled visits beyond 90 days [13].

Multivariable Cox regression was used to assess the association between retention and other variables in the models. The following baseline variables were assessed as potential risk factors for attrition: age, gender, clinical stage, CD4 count at ART start, presence of opportunistic infection, hemoglobin, weight (z-score weight-for-age), height (z-score height-for-age) and education level as children's characteristics; age, education level, gender, religion, profession, civil status and relation with the child as caregivers' characteristics. Variables with p-value ≤0.10 during univariable analysis were considered for inclusion in the multivariable model. Z-scores for weight-for-age and height-for-age were generated using the 2006 WHO growth standards and the zscore06 module in Stata (http://www.ifpri.org/staffprofile/jefleroy).

The complex model was reduced by stepwise backwards elimination approach. Study site was included in the model as a fixed effect. All associations were tested using likelihood-ratio tests.

For variables with less than 10% missing values conditional imputation was used and for variables with at least 10% missing data the missing indicator method (in which a separate category is generated for patients with data missing for a certain predictor). As HEAL Africa Hospital had the highest number of children in care, this site was considered as the reference site. Epi info^TM^ 7.0.9.34 was used to enter data from all the sites by a trained data manager in Kinshasa. The data analysis was conducted using Epi info and Stata 11.

### Ethics statement

The study protocol was approved by the Institutional Review Board (IRB) of the Kinshasa School of Public Health at the University of Kinshasa. All study members were trained in human subject research and good clinical practices.

Due to the nature of the study it was not possible and feasible to obtain informed consent from the patients whose medical charts were abstracted for the study. Data were extracted from routine records, and were analyzed anonymously.

## Results

**Figure 2:**
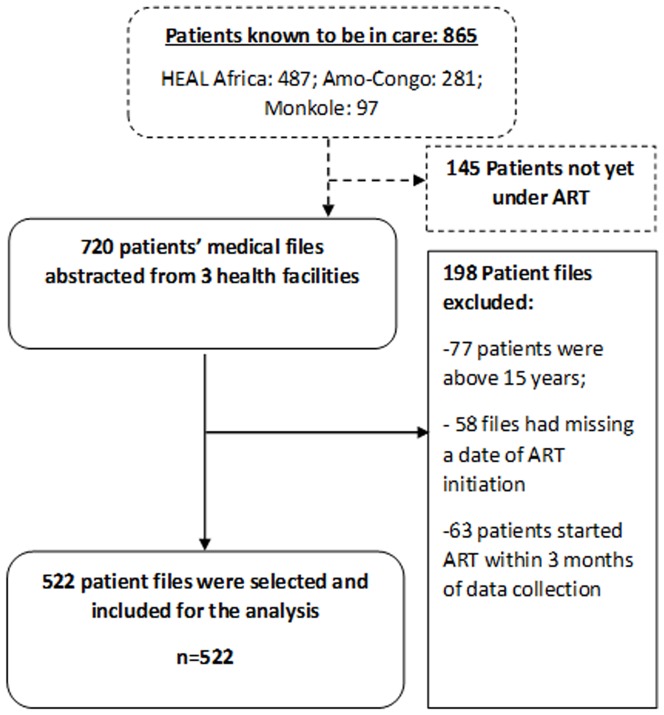
Study flow of participants in three different HIV treatment centers in the Democratic Republic of Congo.

The number of HIV infected children under ART did not reach 250 as was reported by the Amo-Congo and Monkole facilities. Therefore in total only 720 medical files of children on ART were available at the three study sites. Applying the inclusion criteria, 198 patient files were excluded: 77 patients were above 15 years at the moment of data collection, in 58 files the date of ART initiation was missing and 63 patients started ART within 3 months prior to data collection. After this process, 522 patient files were selected and included for the analysis. The details regarding patient flow are presented in **Fig. 2**.

### Children's characteristics (Table 1)

**Table 1:** Children's characteristics in three different programs and sites in the Democratic Republic of Congo.

Children's characteristics	Amo-Congo Kinshasa	Monkole Kinshasa	HEAL Africa Goma	Total
**Total, n (%)**	147 (28.16)	61(11.69)	314(60.15)	522(100.0)
**Age (years), n (%)**				
≤2	47 (31.9)	34 (55.7)	106 (33.8)	187 (35.8)
>2- more	100 (68.1)	27 (44.3)	208 (66.2)	335 (64.2)
**Sex, n (%)**				
Male	76 (51.8)	30 (49.2)	160 (51.0)	266 (51.0)
Female	71 (48.2)	31 (50. 8)	154 (49.0)	256 (49.0)
**Education, n (%)**				
Not yet eligible	47 (31.9)	34 (55.8)	252 (80.2)	333 (63.8)
Kindergarden	14 (9.5)	3 (4.9)	4 (1.3)	21 (4.0)
Primary	72 (48.9)	18 (29.5)	56 (17.8)	146 (27.9)
Secondary	2 (1.4)	0 (0.0)	2 (0.7)	4(0.7)
Missing	12(8.3)	6(9.8)	0(0.0)	18(3.5)
**Weight-for-age z-score, n (%)**				
<−2 SD	19(12.9)	25(41.0)	83(26.4)	127(24.3)
≥−2 SD	85(57.8)	18(29.5)	122(38.9)	225(43.1)
Missing	43(29.3)	18(29.5)	109(34.7)	170(32.6)
**Height-for-age z-score, n (%)**				
<−2 SD	10(6.8)	5(8.2)	76(24.2)	91(17.4)
≥−2 SD	38(25.9)	5(8.2)	109(34.7)	152(29.1)
Missing	99(67.3)	51(83.6)	129(41.1)	279(53.5)
**Clinical stage, n (%)** *				
WHO I	21 (14.3)	5 (8.2)	1 (0.3)	27 (5.2)
WHOII	55 (37.4)	17 (27.9)	4 (1.3)	76 (14.5)
WHO III	64 (43.5)	36 (59.0)	306 (97.5)	406 (77.8)
WHO IV	7 (4.8)	3 (4.9)	3 (0.9)	13 (2.5)
**Drug regimen, n (%)**				
AZT/3TC/NVP	84 (57.1)	49 (80.3)	207 (65.9)	340 (65.1)
3TC/D4T/NVP	36 (24.5)	9 (14.7)	92 (29.3)	137 (26.2)
Other	27 (18.3)	3 (4.9)	15 (4.8)	45 (8.7)
**CD4 cell count** * **(cells/µL), n (%)**				
0–200	49 (33.3)	25 (40.9)	120 (38.3)	194 (37.1)
>200–350	24 (16.3)	7 (11.5)	52 (16.5)	83 (15.9)
>350-more	27 (18.5)	7 (11.5)	89 (28.4)	123 (23.6)
Missing	47(31.9)	22(36.1)	53(16.8)	122(23.4)
**Cotrimoxazole, n (%)**				
No	2 (1.4)	33 (54.1)	12 (3.8)	47 (9.0)
Yes	145 (98.6)	28 (45.9)	302 (96.2)	475 (91.0)

*At the start of antiretroviral treatment.

From 2007 to 20012, 522 HIV positive children were included in the analysis. The mean age of the children was 4.7 years (standard deviation [SD]: 3.3); 80% were in WHO clinical stage 3 or 4 at the start of ART. The first line ART regimen provided to 65% of the children was Zidovudine + Lamivudine + Nevirapine (AZT/3TC/NVP). The proportion of children who did not yet attend school was 43%; 27% attended primary school.

### Care givers' characteristics (Table 2)

**Table 2:** Characteristics of the child's caregiver in three different programs and sites in the Democratic Republic of Congo.

Caregivers' characteristics	Amo-Congo Kinshasa	Monkole Kinshasa	HEAL Africa Goma	Total
**Total, n (%)**	147 (28.16)	61(11.69)	314(60.15)	522 (100.0)
**Type of Caregiver, n (%)**				
Mother	43 (29.3)	20 (32.8)	152 (48.4)	215 (41.2)
Father	31 (21.1)	11 (18.0)	50 (15.9)	92 (17.6)
Grandparent	9 (6.1)	3 (4.9)	22 (7.1)	34 (6.5)
Brother/sister	29 (19.7)	15 (24.6)	47 (14.1)	91 (17.5)
Other	35 (23.8)	12 (19.7)	43 (13.7)	90 (17.2)
**Age (years), n (%)**				
>15–35	32 (21.8)	11 (18.6)	115 (36.6)	158 (30.4)
>35–45	46 (31.3)	21 (35.6)	92 (29.3)	159 (30.6)
>45	63 (42.8)	20 (33.9)	86 (27.4)	169 (32.5)
Missing	6(4.1)	7(11.9)	21(6.7)	34(6.5)
**Sex, n (%)**				
Female	91 (61.9)	33 (54.1)	230 (73.3)	354 (67.8)
Male	56 (38.1)	28 (45.9)	84 (26.7)	168 (32.2)
**Profession, n (%)**				
Housewife	48 (32.7)	15 (28.8)	123 (39.2)	186 (35.6)
Student	10 (6.8)	4 (7.6)	27 (8.6)	41 (7.9)
Employee	35 (23.8)	16 (26.2	60 (19.1)	111 (21.3)
Trader	46 (31.3)	17 (27.8)	88 (28.1)	151 (28.9)
Missing	8(5.4)	9(14.8)	16(5.1)	33(6.3)
**Religion, n (%)**				
Catholic	44 (29.9)	14 (22.9)	109 (34.7)	167 (32.0)
Protestant	11 (7.5)	14 (22.9)	102 (32.5)	127 (24.3)
Independent	65 (44.2)	20 (32.9)	55 (17.5)	140 (26.8)
Other	22 (15.0)	8 (13.1)	36 (11.5)	66 (12.6)
Missing	5(3.4)	5(8.2)	12(3.8)	22(4.2)
**Education, n (%)**				
Primary and less	26 (17.7)	12 (19.7)	128 (40.8)	166 (31.8)
Secondary	83 (56.5)	27 (44.2)	132 (42.1)	242 (46.4)
Higher education	35 (23.8)	10 (16.4)	39 (12.3)	84 (16.1)
Missing	3(2.0)	12(19.7)	15(4.8)	30(5.7)
**Marital status, n (%)**				
Not maried	25 (17.0)	8 (13.1)	32 (10.1)	65 (12.5)
Married (Poly and monogamous)	53 (36.1)	15 (24.5)	150 (47.8)	218 (41.8)
Widowed	37 (25.2)	11 (18.0)	80 (25.5)	128 (24.5)
Missing	32(21.7)	27(44.3)	52(16.6)	111(21.3)

In 51%, the children's caregivers were direct parents (either mother of father). Among the caregivers, 51% were male and the mean age of the caregivers was 42 years (SD: 13). Those who had a formal job represented 53% of the caregivers, 66% had at least the secondary school education. 42% of the caregivers were married, 62% belonged either to the catholic or protestant church, and 37% to the independent church.

### Retention

**Figure 3:**
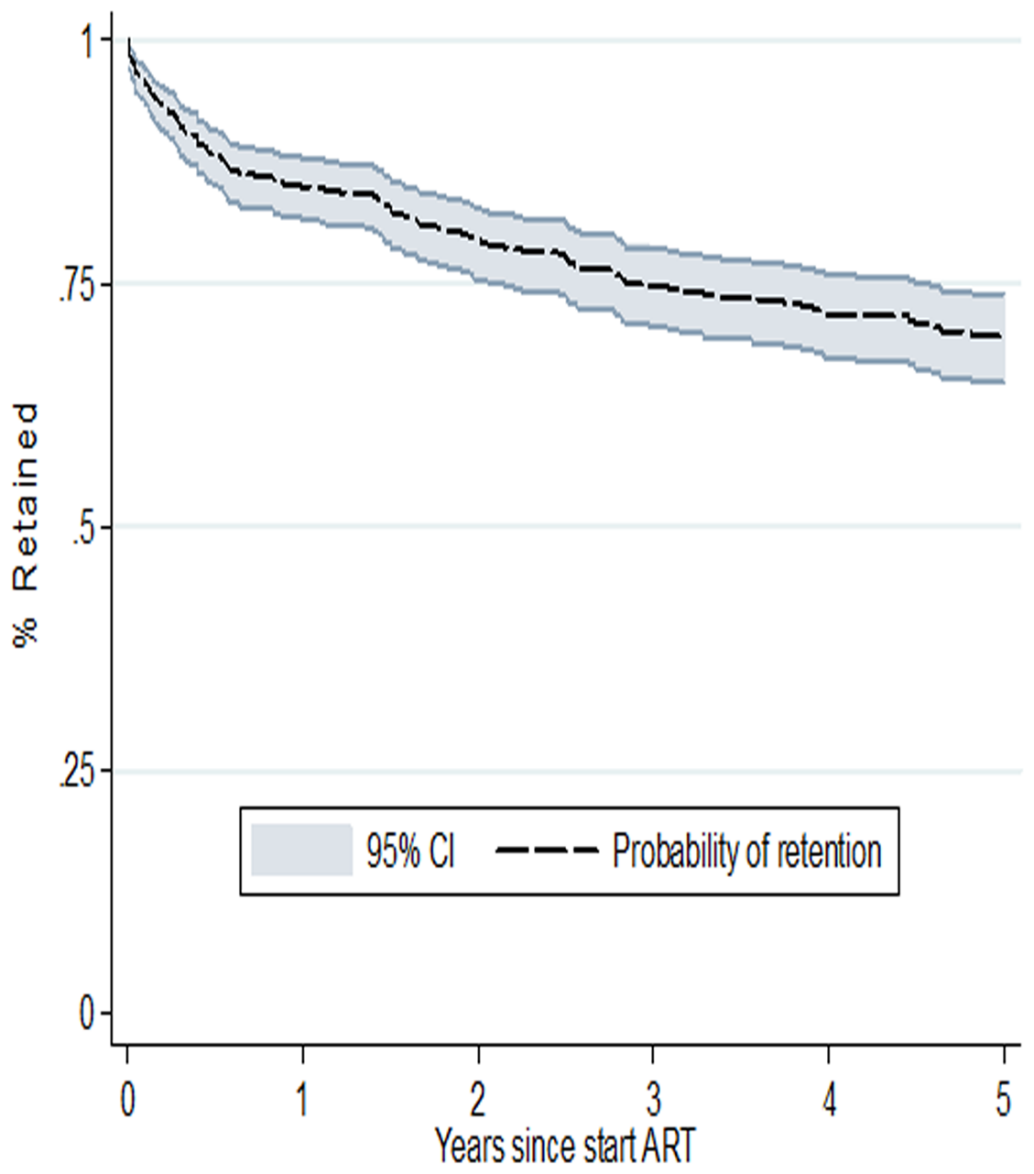
Overall retention rates for children on ART in three different HIV treatment centers in the Democratic Republic of Congo.

**Figure 4:**
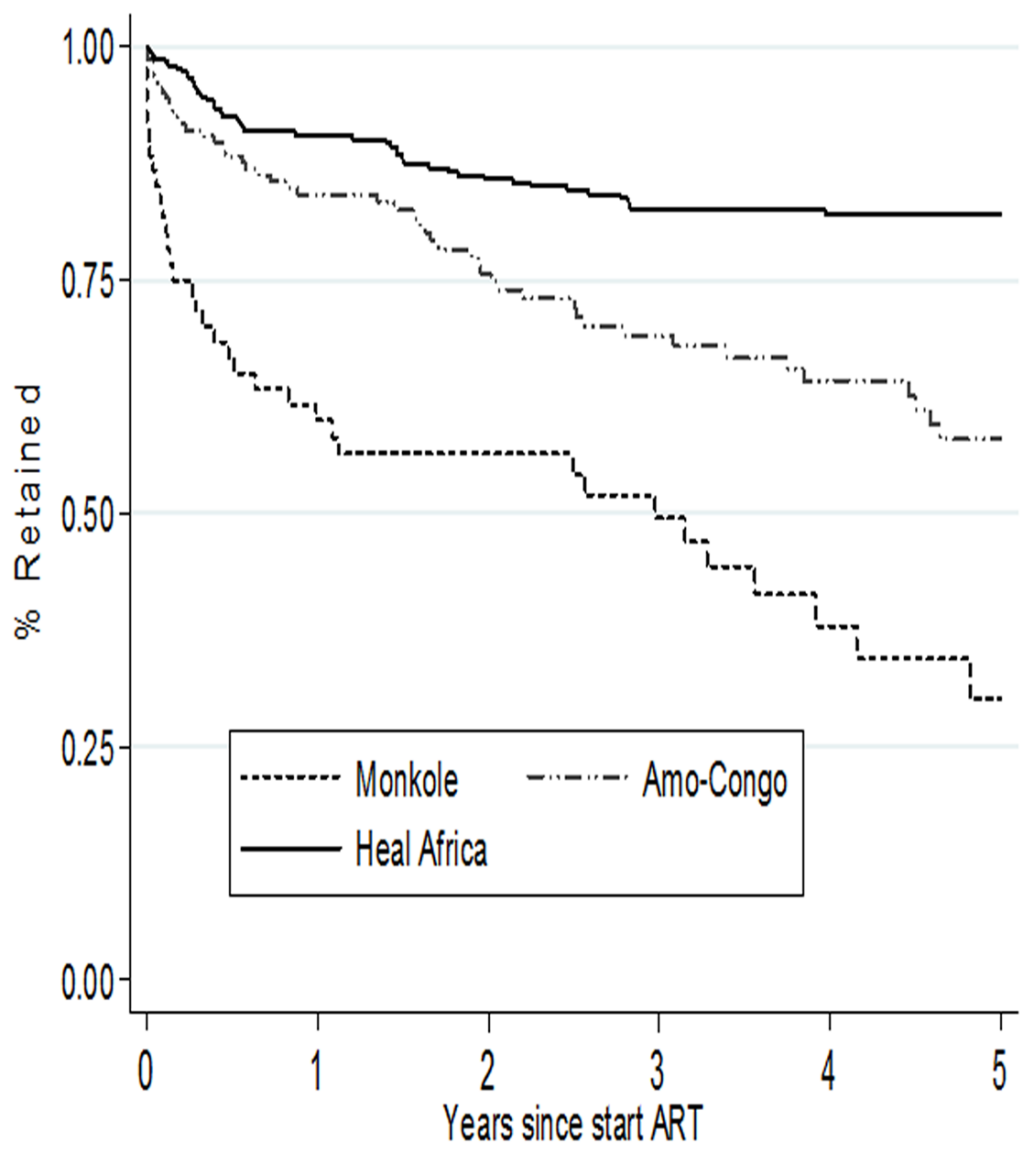
Retention rates for children on ART by facility in three different programs and sites in the Democratic Republic of Congo.

The overall retention probabilities are presented in **Fig. 3**. Overall retention rates were 88% (95% CI [95% confidence interval]: 85%–91%) at 6 months, 85% (95% CI: 81%–87%) at one year, 79% (95%CI: 75%–83%) at two years and 74% (95% CI: 70%–78%) at 3 years. The overall median time in the ART program was 3 years (IQR [inter quartile range]: 1.3–5.0), with site-specific median times of 2.5 years (IQR: 1.3–4.6) at AMO-Congo Kasavubu, 1.5 years (IQR: 1.2–3.5) at the Monkole facility and 3.5 (IQR: 1.5–5.0) years at the HEAL Africa facility in Goma. Site-specific retention rates were 88%, 67% and 92% at 6 months; 84%, 59% and 90% at 12 months and 76%, 56% and 86% at 24 months, respectively for Amo-Congo/Kasa-vubu, Monkole facility and HEAL Africa (**Fig. 4**).

Out of the 522 HIV infected children, 109 (20.8%) were lost from the ART program: 22 (20%) had died, 87 (80%) were lost to follow-up and, 4 (4%) were transferred to other ART programs.

### Risk factors for attrition (Table 3)

**Table 3:** Risk factors for attrition in three different programs and sites in the Democratic Republic of Congo.

Riskfactor		Participants N(%)	Unadjusted HR (95% CI)	Adjusted HR (95% CI)	P-value
**Study site, n (%)**	HEAL Africa	314 (60.5)	1	1	<0.001
	Amo-Congo	147 (27.9)	2.29 (1.54–3.42)	2.15 (1.35–3.40)	
	Monkole	61 (11.6)	5.14 (3.32–7.94)	4.77 (2.90–7.85)	
**Initial age of Child (years), n (%)**	>2 years	334(66.2)	1	1	<0.001
	≤2 years	170(33.8)	2.41(1.71–3.40)	2.16 (1.48–3.16)	
**Initial CD4 cell count of child (cells/µL), n (%)**	≥350-more	121 (23.4)	1	1	0.039
	>200–<350	83 (15.9)	3.12 (1.55–6.27)	2.87 (1.36–6.07)	
	>0–200	193 (37.2)	3.29 (1.77–6.11)	2.70 (1.40–5.21)	
	Missing	122 (23.5)	3.80 (1.98–7.29)	2.24 (1.11–4.51)	
**Education of child, n (%)**	Primary or less	138 (27.6)	1	/	/
	Secondary school	149 (29.7)	2.74 (1.56–4.82)		
	Not yet eligible	214 (42.7)	2.90 (1.70–4.95)		
**Religion of caregiver, n (%)**	Catholic	166 (33.4)	1	1	0.003
	Protestant	144 (29.0)	0.78 (0.44–1.36)	1.90 (0.51–1.59)	
	Independant/other	187 (37.6)	2.07 (1.34–3.21)	1.86 (1.19–2.90)	

HR: Hazard Ratio; CI: Confidence Interval;

P-value from Cox-regression model adjusted for all factors in final model, including site as a fixed effect.

**Figure 5:**
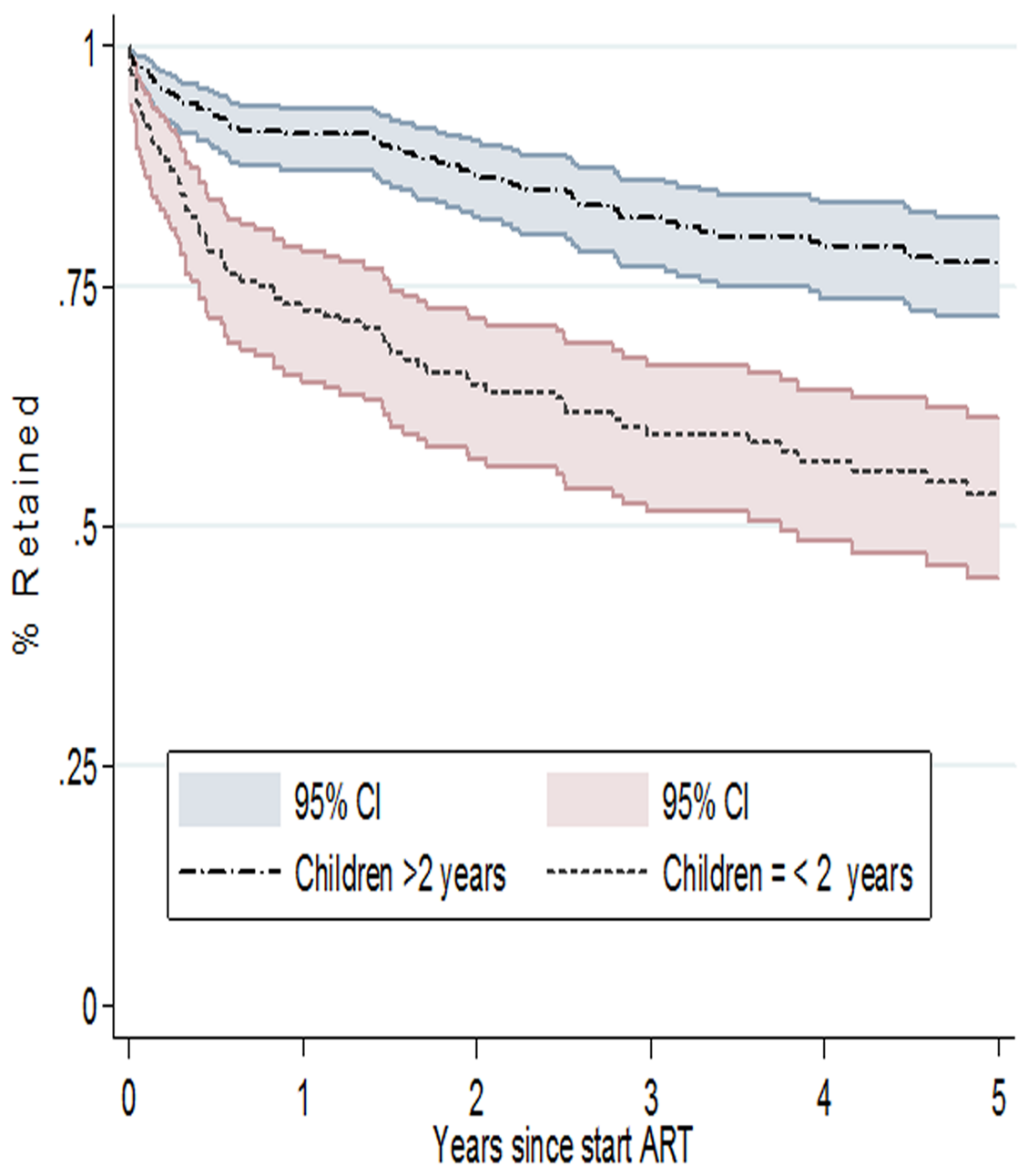
Retention rates for children on ART by age categories in three different programs and sites in the Democratic Republic of Congo.

The following variables were considered in the multivariable model: study site, the child's age, CD4 count, education level and uptake of Cotrimoxazole; type, gender and religion of caregiver. In the final model four variables remained associated with children's attrition from ART programs: study site, the child's age and CD4 count at ART start and religion of the caregiver. Patients enrolled in the Monkole facility (aHR: 4.77, 95% CI: 2.90–7.85) were likely to experience higher risk of attrition compared to the patients enrolled in care at the HEAL Africa hospital. Children ≤2 years age (aHR: 2.16, 95% CI: 1.48–3.16) experienced higher attrition compared to older children (**Fig. 5**). Children with a CD4 cell count ≤200/µL (aHR: 2.70, 95% CI: 1.40–5.21) and those with a CD4 cell count between >200–<350/µL (aHR: 2.87, 95% CI: 1.36–6.07) were likely to experience higher risk of attrition compared to children with CD4 count ≥350/µL.

Children whose caregivers were affiliated to the independent church (aHR: 1.86; 95% CI: 1.19–2.90) were more likely to experience attrition compared to those whose caregivers were affiliated to the catholic and protestant church.

### Risk factors for loss to follow up and death (Table 4)

**Table 4:** Risk factors associated with LTFU and death among children in three study sites.

		Loss to follow up		Reported deaths	
Riskfactor		Unadjusted HR (95% CI)	Adjusted HR (95% CI)	Unadjusted HR (95% CI)	Adjusted HR (95% CI)
**Study site**	HEAL Africa	1	1	1	1
	Amo-Congo	2.28 (1.49–3.50)	2.06(1.27–3.36)	9.52(4.15–21.83)	7.22(2.92–17.84)
	Monkole	4.31 (2.69–6.92)	3.41(1.98–5.86)	12.63(5.14–30.99)	7.49(2.84–19.77)
**Initial age of Child (years)**	>2 years	1	1	1	1
	≤2 years	2.20(1.52–3.18)	1.89(1.25–2.85)	1.80(1.03–3.14)	2.78(1.19–6.51)
**Initial CD4 cell count of child (cells/µL)**	≥350-more	1	1	1	/
	>200–<350	3.81 (1.74–8.33)	3.80 (1.64–8.77)	1.47 (0.55–3.92)	
	>0–200	3.70 (1.82–7.51)	3.13 (1.46–6.68)	1.14 (0.48–2.69)	
	Missing	4.29 (2.04–9.00)	2.83 (1.27–6.29)	2.64 (1.15–6.04)	
**Education of child**	Primary or less	1	/	1	/
	Secondary school	2.47 (1.39–4.38)		4.37 (1.64–11.61)	
	Not yet eligible	2.31 (1.34–3.97)		2.48 (0.92–6.66)	
**Religion of caregiver**	Catholic	1	1	1	/
	Protestant	0.85 (0.46–1.54)	1.02 (0.55–1.88)	0.33 (0.10–1.01)	
	Independant/other	2.10 (1.31–3.37)	1.79 (1.10–2.90)	2.02 (1.04–3.90)	

HR: Hazard Ratio; CI: Confidence Interval.

In multivariate analysis, four variables remained associated with loss to follow up: study site, the child's age and CD4 count at ART start and religion of the caregiver. Children enrolled in the Monkole facility (aHR: 3.41, 95% CI: 1.98–5.86) were likely to experience higher risk of loss to follow up compared to the patients enrolled in care at the HEAL Africa hospital. Children ≤2 years age (aHR: 1.89, 95% CI: 1.25–2.85.) experienced higher risk of loss to follow up compared to older children. Children with a CD4 cell count ≤200/µL (aHR: 3.13, 95% CI: 1.46–6.68) and those with a CD4 cell count between >200–<350/µL (aHR: 3.80, 95% CI: 1.64–8.77) were likely to experience higher risk of loss to follow up compared to children with CD4 count ≥350/µL. Children whose caregivers were affiliated to the independent church (aHR: 1.79; 95% CI: 1.10–2.90) were more likely to experience attrition compared to those whose caregivers were affiliated to the catholic and protestant church.

Two variables remained associated with death: study site and the child's age. Patients enrolled in the Monkole facility (aHR: 7.49, 95% CI: 2.84–19.77) were likely to experience higher risk of death compared to the patients enrolled in care at the HEAL Africa hospital. Children ≤2 years age (aHR: 2.78 95% CI: 1.19–6.51) experienced higher risk of death compared to older children.

## Discussion

Retention rates varied widely across the study facilities: between 93% and 67% at 6 months; 90% and 59% at 12 months and 86% to 56% at 24 months. The highest retention rate was found in Goma, which can be described as a mixture of a peri-rural and rural setting. The community support organization active in this rural area may have played a role in retaining ART patients as was also observed in other settings [14]. Our findings corroborate also with other studies that report higher retention rates in rural areas compared to urban areas [4], [15]–[20].Retention rates among children on ART in our study were high compared to another study conducted among adults in the DRC, where retention rates of 81%, 75%, 65% and 57%, respectively at 6 months, one year, two years and three years were found [21]. Somehow these better results among children highlight the low quality of care level for patients with HIV infection in the DRC in general.

In the DRC the coverage in HIV care services is amongst the lowest in the world. PMTCT interventions remain the entry point for pediatric care treatment in DRC, but PMTCT coverage is very low (13%) [2]. Also coverage of family planning in DRC is very low, only 6% of women of childbearing age have access to family planning [22]. The ART coverage in adults is less than 20%, with huge disparities across the country and alarming figures in the rural areas. ART coverage is even more dramatic in children: 9% in 2012 [2]. As a consequence of this low coverage of family planning, PMTCT and ART the number of HIV infected children is increasing. Advocacy and increased commitment from government and international organizations is needed to increase services for family planning, PMTCT and ART coverage [2]. HIV services should be implemented in all health facilities allowing nurses to provide the ARV.

Retention rates were significantly higher in HIV specialized facilities like HEAL Africa and Amo-Congo/Kasa-vubu compared to Monkole which is a general hospital and which offers a packet of integrated health services. In the DRC, most of public facilities are general health facilities that do not pay special attention to a complex health issue such as HIV. Poor quality of health services is associated with high attrition rates [23].

Patients with a CD4 cell count <350/µL were more likely to experience attrition compared to those with higher CD4 counts. We should be cautious about the interpretation of these findings. More than 10% of data on CD4 cell count was missing and the CD4 cell count percentage (to determine the eligibility for ART in children) was not available for most of the children. We speculate that children with a CD4 cell count <350/µL were at higher risk of mortality and lost to follow up as reported in other studies where attrition was associated with advanced disease stage [4]–[6].

Younger children (≤2 years) were also more likely to experience attrition. These findings corroborate with others studies conducted in developing countries [5]–[6], [24]. The children whose caregivers were affiliated to the independent churches were more likely to experience attrition compared to those whose caregivers were affiliated to the catholic church or protestant church. These independent churches often promise miracle healing. Therefore patients affiliated to these churches are more at risk for attrition. These findings corroborate with others studies where independent church members appear to have lower uptake of health services [25], [26]. Given the impact of religion on people in the DRC, collaboration with religious leaders could help to convince caregivers about the importance not to stop the ART. Specific training sessions for religious leaders about this topic should be considered.

Reinforcement of community interventions such as the “Mentor Mothers (MM)” and “Community ART groups (CAG)” is critical [27], [28]. MM approach consists of using HIV positive peer mothers to support, educate, and empower other pregnant women, new mothers and their partners [27]. CAG are peer support groups that provide psychosocial support to HIV-positive women, men, and children in their own community [28]. Each CAG comprises between 6 to 12 members and keeps linkage with the health facility in order to receive ARVs and clinical support as needed. CAG members organize themselves to meet and provide peer support without involvement of the health providers. These support groups also play a key role in defaulter tracing and encouraging attendance at health facility appointments. The CAG approach helps to reduce the frequency of drugs' collection from the pharmacy and therefore the cost for traveling to the clinic. One patient (volunteer) travels to the clinic to collect ARVs for other members of the CAG. The CAG strategy was shown to improve significantly patients' retention and adherence [14], [28].

Our study has several limitations. As in most retrospective studies, some important information was not available in the medical charts e.g. weight, height and CD4%. Moreover, we were unable to assess the influence of the caregivers HIV status because this information was only available in a few of them. Other studies have shown that an HIV positive status of the caregiver may have a negative impact on the children's retention [28].

Our study was performed in three non-public facilities. We speculate that the support provided to these facilities had a positive impact on the quality of data and services. Results may be worse in public facilities in the DRC where the support is scarce.

In conclusion, attrition remains a challenge for pediatric HIV positive patients in ART programs in the DRC. In addition, the low coverage of pediatric treatment and the delayed start of ART exacerbate the situation of pediatric HIV/AIDS. Whilst moving towards the new 2013 WHO guidelines [29], serious commitment, in terms of government's political will and more support from the international donors, is needed to increase access to ART and retention in ART programs.
